# Incorporating platelet-to-white blood cell ratio into survival prediction models for intracerebral hemorrhage: a nomogram approach

**DOI:** 10.3389/fneur.2024.1464216

**Published:** 2024-10-10

**Authors:** Jiake Xu, Xing Wang, Wei Chen, Meng Tian, Chao You

**Affiliations:** ^1^Department of Neurosurgery, Neurosurgery Research Laboratory, West China Hospital, Sichuan University, Chengdu, Sichuan, China; ^2^West China Brain Research Centre, West China Hospital, Sichuan University, Chengdu, Sichuan, China

**Keywords:** intracerebral hemorrhage, Medical Information Mart for Intensive Care-IV database (MIMIC-IV database), nomogram, platelet, white blood cell

## Abstract

**Background:**

Predicting long-term survival in intensive care unit patients with intracerebral hemorrhage (ICH) is crucial. This study aimed to develop a platelet-to-white blood cell ratio (PWR) incorporated nomogram for long-term survival prediction.

**Methods:**

A retrospective analysis was conducted on 1,728 ICH patients in the MIMIC-IV 2.2 database. The independent prognostic value of PWR for 1-year mortality was assessed. A nomogram was developed using LASSO and Cox regression to predict 1-year survival, incorporating PWR and other factors. The performance of the nomogram was evaluated through calibration curves, area under the curve, Delong test, net reclassification index, integrated discrimination improvement, and decision curve analysis.

**Results:**

The nomogram, which included age, weight, Glasgow Coma Scale (GCS) score, mechanical ventilation, glucose, red blood cell (RBC) count, blood urea nitrogen (BUN), and PWR, showed good predictive performance for 1-year survival. The C-index was 0.736 (95% CI = 0.716–0.756) for the training set and 0.766 (95% CI = 0.735–0.797) for the testing set. Higher age and ventilation increased mortality risk, while higher weight, GCS score, RBC count, and PWR decreased risk. The nomogram outperformed conventional scores.

**Conclusions:**

A nomogram incorporating PWR as a prognostic factor accurately predicts long-term survival in ICH patients. However, validation in large-scale multicenter studies and further exploration of biomarkers are needed.

## Introduction

Intracerebral hemorrhage (ICH) accounts for about 15% of all strokes and imposes a significant global burden in terms of disability-adjusted life years, often leading to various levels of functional impairment (1). Unlike ischemic strokes, there are currently no effective clinical treatments to prevent neuronal damage or promote neural repair for ICH (2, 3). Therefore, it is essential to fully understand the severity of ICH prognosis early on and identify risk factors that lead to poor outcomes in ICH patients to prepare for prevention and treatment.

Evaluating disease severity through scoring systems is beneficial for assessing patient prognosis and adjusting treatment plans. Many ICH scoring systems have been developed to date, primarily combining basic patient information with parameters obtained from computed tomography (CT) and neurological examinations (4, 5). It is widely accepted that the pathophysiological process of ICH consists of two stages: primary injury and more complex secondary injury. Inflammation is closely related to the progression of secondary injury and directly affects treatment outcomes (6). Current research suggests that inflammation induced by ICH manifests as changes in peripheral blood immune cells (7), with many inflammation markers related to disease progression, such as platelets, white blood cell (WBC) count, and neutrophils, easily obtainable through routine laboratory tests. Moreover, ratios of certain variables, such as the neutrophil-to-lymphocyte ratio (NLR), provide more stable predictive performance compared to single variables and have found widespread application in clinical settings (8).

The platelet-to-white blood cell ratio (PWR), a newly discovered inflammatory biomarker similar to NLR, has shown excellent predictive performance in ischemic stroke patients, although its role as an independent prognostic factor in ICH patients remains to be confirmed (9–11). Given its unique characteristics, PWR reflects both inflammation and coagulation processes, which are key factors in the pathophysiology of ICH. Unlike other indicators such as NLR, PWR provides a more comprehensive view by combining information on platelet function and immune response, making it a robust indicator of patient status. Additionally, PWR is easily obtainable through routine laboratory tests, making it a practical and accessible marker for clinical use. Incorporating these readily available laboratory markers into scoring systems may help create more convenient and accurate prediction models (12). Additionally, while many scoring systems have been developed, most only predict in-hospital or one-month mortality rates, with few studies focusing on longer-term outcomes such as 1-year prognoses (5).

In this study, we first examined the role of the PWR in predicting ICH mortality. To more accurately assess the prognosis of ICH patients, we developed a prediction model incorporating PWR using the lasso method and multivariate regression analyses and constructed corresponding nomograms for predicting 3-month, 6-month, and 1-year survival. By validating the nomograms, we evaluated the accuracy and reliability of this model in predicting ICH survival. This study aims to provide clinicians with a more effective tool for assessing the prognosis of hemorrhagic stroke patients, thereby enabling the development of more appropriate treatment plans.

## Methods

### Data source

We obtained patient data from the publicly available Medical Information Mart for Intensive Care (MIMIC-IV, Version 2.2) database (13). Developed by the Massachusetts Institute of Technology Lab for Computational Physiology, MIMIC-IV provides comprehensive data on 315,460 inpatients from 2008 to 2019. As all personal information has been deidentified using random codes to replace patient identification, ethical approval or informed consent was not required to access the database, ensuring patient privacy. The authors (Jiake Xu and Wei Chen) have been authorized to access the database.

### Patients selection

**Figure 1:**
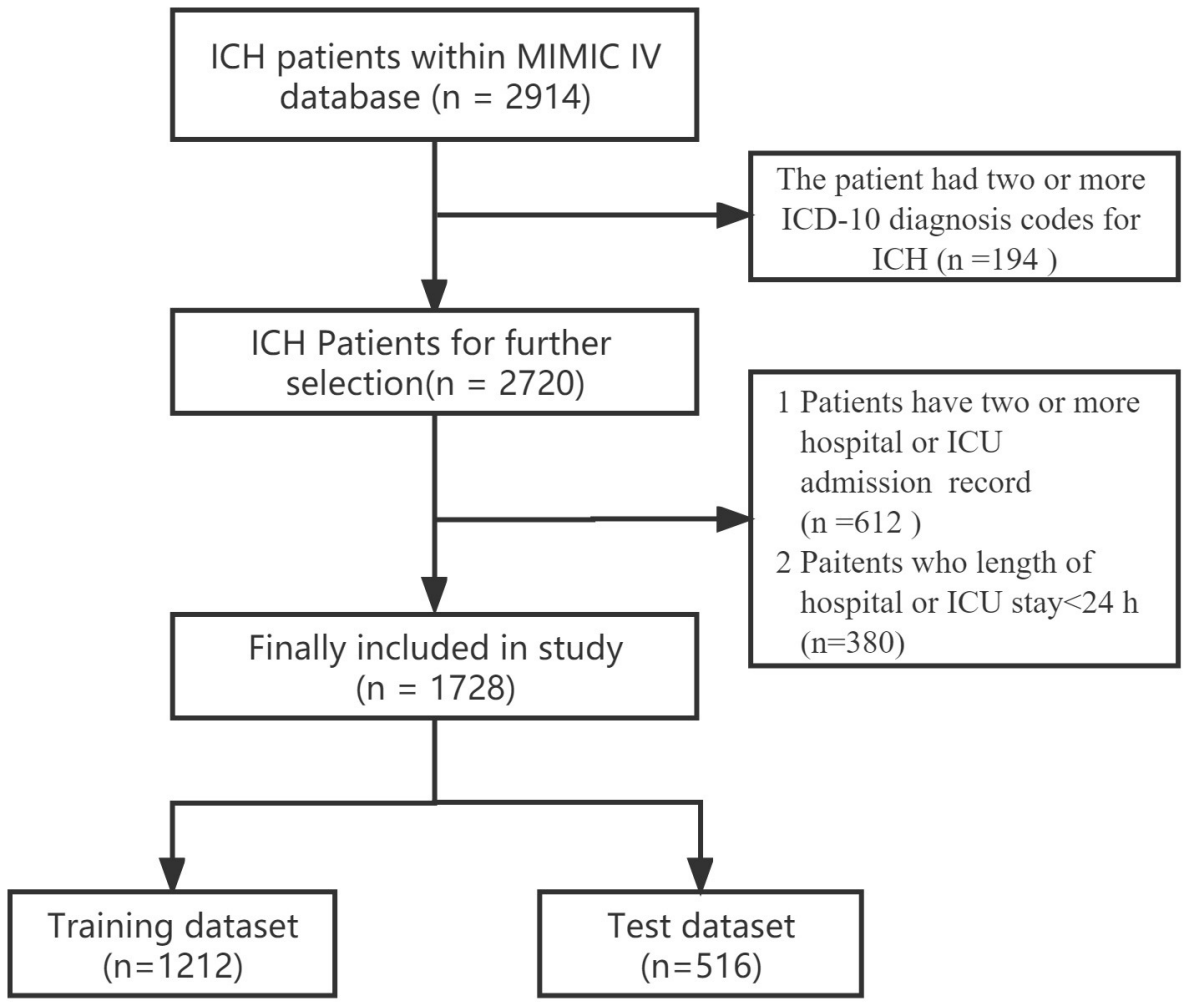
The study participant flow diagram.

The inclusion criteria for this study comprised patients with ICH, identified using ICD-9 codes 431, and ICD-10 codes I61-I62. Samples were excluded if patients were younger than 18 years old or stayed in the hospital for <24 h. For patients admitted to the hospital more than once, only the initial admission data were used. Further details regarding the selection process are provided in Figure 1.

### Data extraction

Structured Query Language (SQL) with DBeaver (version 22.3.4) was used to extract baseline characteristics, comorbidities, laboratory variables, and other data within the first 24 h after intensive care unit (ICU) admission from the MIMIC-IV 2.2 database. Baseline characteristics included age, gender, weight, ethnicity, and whether the patient smokes or has alcohol abuse issues. Severity at admission was measured by the Glasgow Coma Scale (GCS), Acute Physiology Score III (APSIII), and Oxford Acute Severity of Illness Score (OASIS). Comorbidities included heart disease, chronic pulmonary disease, rheumatic disease, diabetes, renal disease, malignant cancer, liver disease, and hypertension. The maximum values for the first 24 h of laboratory variables were collected after admission, including hemoglobin, platelet count, WBC, red blood cell (RBC) count, blood urea nitrogen (BUN), creatinine, glucose, prothrombin time (PT), and activated partial thromboplastin time (APTT). For patients with available imaging data, hematoma volume, presence of intraventricular hemorrhage, and hematoma origin were extracted to calculate the ICH Score. The PWR is defined as the ratio of platelet count to WBC. For missing data in continuous variables, the random forest imputation method was used to fill in the gaps. Variables with missing values ≥20% were excluded.

### Outcome

The primary outcome in this study was 1-year survival. Secondary outcomes included 3- and 6-month survival.

### Statistical methods

Data processing and analysis were conducted using R software (4.2.2). Descriptive statistics were utilized to summarize continuous variables. The Mann-Whitney test was applied to non-normally distributed continuous variables, while the Chi-square test was employed for binary data. Patients were randomly divided 7:3 into a training set and a testing set. In the training set, logistic regression was used to evaluate the predictive value of PWR as an independent predictor of survival and ICU-related complications in patients with ICH. The optimal cutoff value of PWR was identified using receiver operating characteristic (ROC) curves. Variable selection was performed using LASSO regression, and a multivariable Cox regression model was used to create a nomogram of independent predictors. The model's performance was assessed using calibration curves, area under the curve (AUC), Delong test, net reclassification improvement (NRI), integrated discrimination improvement (IDI), decision curve analysis (DCA), and Kaplan-Meier curves in both the training and testing sets.

## Results

### Population and baseline characteristics

In the MIMIC-IV version 2.2 database, a total of 2,914 ICH cases were identified among patients aged 18 years and older. We focused on 1,728 patients with ICH after excluding duplicate records, multiple admissions, and short hospital/ICU stays. These patients were then randomly divided into the training set (*n* = 1,212) and testing set (*n* = 516). The study flow diagram is shown in Figure 1, and the baseline characteristics of the training set and testing set patients are detailed in the supporting material (Supplementary Table 1). Among the patients in the training set, a total of 708 patients survived (Supplementary Table 2). Factors such as older age, certain medical conditions, and ICU-related complications (including infection, kidney failure, and hydrocephalus) were associated with higher mortality rates in the training set. Additionally, significant differences in laboratory indicators, including the PWR, were observed between the survival and non-survival groups.

### PWR as an independent prognostic factor

The results in Table 1 show the relationship between PWR and the mortality and complications of ICH patients after adjusting for all relevant covariates (including age, weight, ethnicity, gender, underlying diseases, and laboratory indicators). After adjusting for logistic regression, PWR showed significant associations with 3-month, 6-month, and 1-year mortality rates, as well as the development of hydrocephalus, nosocomial infections, and kidney failure. For 1-year mortality, ROC analysis indicated that PWR had a C-index of 0.6 and an AUC of 0.603 (95% CI, 0.571–0.636), with a sensitivity of 39.1% and a specificity of 80.93% at an optimal cutoff value of 14.846 (Supplementary Figure 1). Based on the cutoff value, the 1,212 patients were divided into a low PWR group (PWR ≤ 14.846, *n* = 327) and a high PWR group (PWR > 14.846, *n* = 885), as shown in Supplementary Table 3. Compared with patients with PWR > 14.846, patients in the PWR ≤ 14.846 group had a significantly longer ICU stay (4.88 vs. 3.62 days, *p* < 0.001) and faced higher 1-year mortality (58.7 vs. 35.3%, *p* < 0.001).

**Table 1:** Comparison of unadjusted and risk-adjusted outcomes by PWR status.

**Outcomes**	**Unadjusted**	***p*-value**	**Adjustment**	***p*-value**
	**OR (95% CI)**		**OR (95% CI)**	
**Mortality**				
3 M mortality	0.958 (0.944, 0.972)	<0.001	0.967 (0.951, 0.983)	<0.001
6 M mortality	0.963 (0.949, 0.976)	<0.001	0.970 (0.955, 0.985)	<0.001
1 Y mortality	0.974 (0.961, 0.986)	<0.001	0.98 (0.966, 0.995)	0.008
**Complication**				
Cerebral edema	0.986 (0.974, 0.998)	0.02	0.987 (0.975, 1.00)	0.056
Hydrocephalus	0.967 (0.949, 0.985)	<0.001	0.979 (0.959, 0.998)	0.034
Nosocomial infections	0.965 (0.952, 0.977)	<0.001	0.981 (0.968, 0.995)	0.006
Kidney failure	0.945 (0.925, 0.966)	<0.001	0.957(0.933, 0.980)	<0.001

### Construction of nomogram

**Figure 2:**
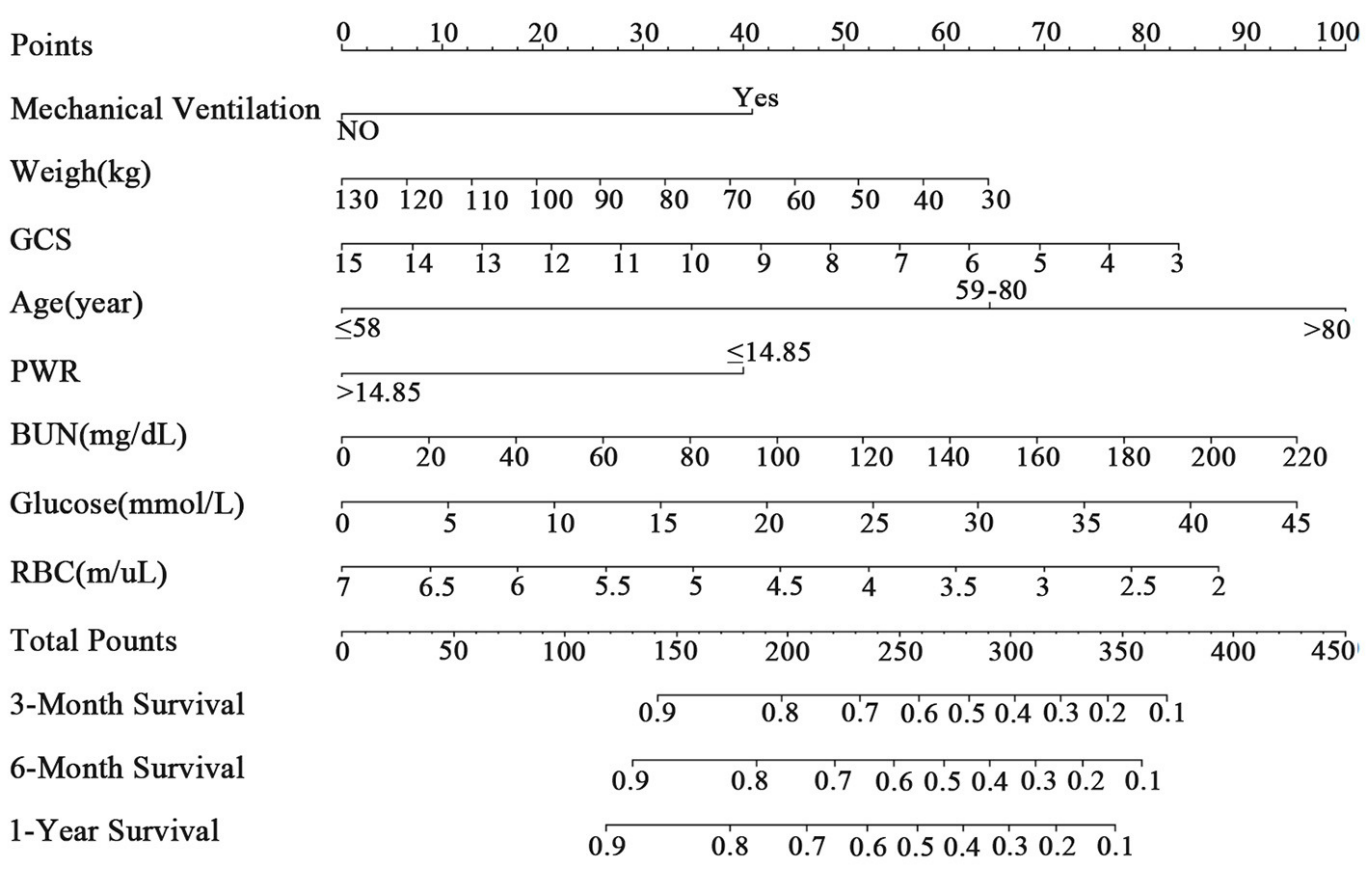
The nomogram for patients with ICH. GCS, Glasgow Coma Scale; PWR, platelet-to-white blood cell ratio; BUN, blood urea nitrogen; RBC, red blood cell.

In this study, we used LASSO analysis to identify the most valuable predictive variables, employing the Lambda.1se strategy to determine the optimal Lambda value (Supplementary Figure 2). We selected nine potential predictor variables based on significant coefficient estimates from the LASSO regression analysis, indicating a strong association with the response variable. Two of these variables represented different age groups, which we combined to reduce the number of input variables to eight for the final model. These variables included weight, GCS, mechanical ventilation, RBC, BUN, glucose, PWR, and age. We performed Cox regression analysis using the backward method to identify the most significant predictors of the outcome variable for 1-year survival in patients (Table 2). Based on these results, we constructed a clinical prediction model and presented it as a nomogram to evaluate patient survival rates (Figure 2), with an example shown in Supplementary Figure 3.

**Table 2:** Cox regression analysis of significant predictors for 1-year survival in patients.

**Variables**	**HR**	**95% CI**	***p*-value**
Age group (59–80)	2.390	1.809–3.157	<0.001
Age group (>80)	3.854	2.879–5.160	<0.001
Weight	0.991	0.986–0.997	0.001
GCS	0.911	0.887–0.935	<0.001
Mechanical Ventilation	1.734	1.421–2.115	<0.001
RBC	0.790	0.681–0.917	0.002
BUN	1.006	1.002–1.010	0.003
Glucose	1.029	1.009–1.049	0.004
PWR	0.583	0.483–0.703	<0.001

GCS, Glasgow Coma Scale; PWR, platelet-to-white blood cell ratio; BUN, blood urea nitrogen; RBC, red blood cell.

### Nomogram evaluation

**Figure 3:**
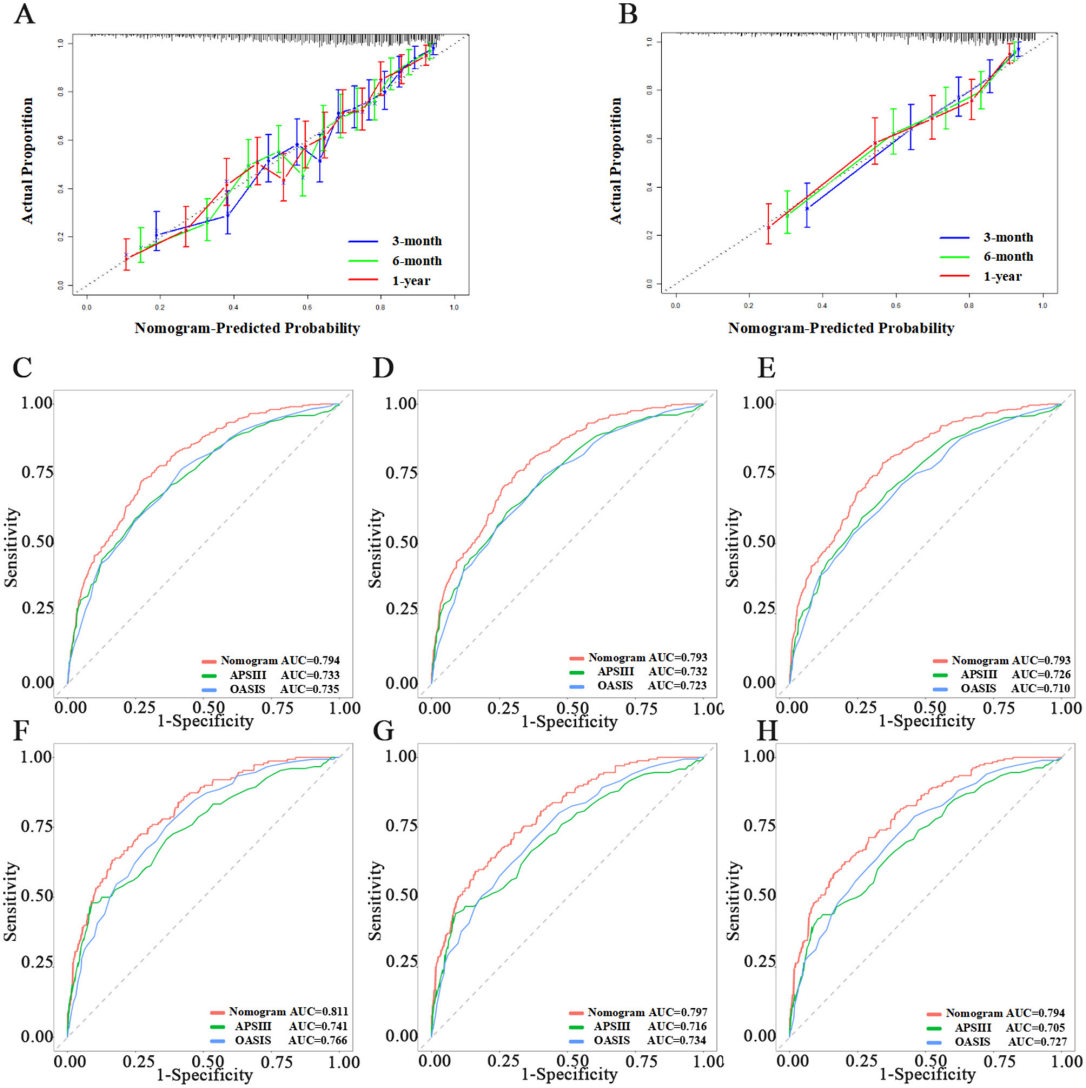
Evaluation of the nomogram. **(A, B)** Calibration curves of Nomogram in the training set and testing set, respectively. **(C–E)** The ROC curves of 3-month, 6-month, and 1-year in the training set, respectively. **(F–H)** The ROC curves of 3-month, 6-month, and 1-year in the testing set, respectively.

We assessed the predictive model using calibration curves. The curves for 3, 6 months, and 1 year closely matched the 45-degree line, demonstrating high accuracy and reliability of the model's predicted survival probabilities compared to observed survival rates (Figures 3A, B). The C-index for the 1-year survival rate was calculated for both the training and testing sets, yielding 0.736 (95% CI = 0.716–0.756) for the training set and 0.766 (95% CI = 0.735–0.797) for the testing set.

We then used ROC, Delong tests, NRI, and IDI to compare the nomogram with traditional scoring systems. In the training set, the AUCs for the nomogram, APSIII, and OASIS scores for the 3-, 6-month, and 1-year survival rates were as follows: nomogram, 0.794, 0.793, and 0.793; APSIII, 0.733, 0.732, and 0.726; and OASIS, 0.735, 0.723, and 0.710. In the testing set, the AUCs for the nomogram, APSIII, and OASIS scores for the 3-, 6-month, and 1-year survival rates were: nomogram, 0.811, 0.797, and 0.794; APSIII, 0.741, 0.716, and 0.705; and OASIS, 0.766, 0.734, and 0.727. The nomogram consistently outperformed APSIII and OASIS scores, as indicated by higher AUC values (Figures 3C–H). The DeLong test confirmed the nomogram's superiority over APSIII and OASIS with statistically significant differences (*p* < 0.001) in both the training and testing sets. Specifically, the categorical NRI for the nomogram compared to APSIII was 0.351 (95% CI: 0.269–0.423) in the training set and 0.419 (95% CI: 0.315–0.527) in the testing set, showing significant improvements in the ability to correctly classify patients into risk categories. Similarly, the continuous NRI was 0.543 (95% CI: 0.455–0.649) in the training set and 0.564 (95% CI: 0.438–0.713) in the testing set, reflecting the nomogram's enhanced discriminative performance. The IDI values for the nomogram compared to APSIII were 0.103 (95% CI: 0.074–0.147, *p* < 0.001) in the training set and 0.132 (95% CI: 0.075–0.201, *p* < 0.001) in the testing set, further confirming the superior ability of the nomogram to differentiate between survival and non-survival. When compared to OASIS, the nomogram also showed significant improvements in both NRI and IDI metrics (Table 3 and Supplementary Table 4). Regarding the traditional ICH score, only 124 patients had complete imaging data available. In this imaging testing dataset, the Delong test confirmed that the nomogram performed as well as the traditional ICH score (*p* > 0.05; Supplementary Table 5; Supplementary Figures 4A, B).

**Table 3:** Evaluation of the nomogram for 1-year survival in the training and testing sets.

**Test**	**Training set**			**Testing set**		
	**Estimate (*****Z*** **for DeLong test)**	**95% CI**	***p*** **-value**	**Estimate (*****Z*** **for DeLong test)**	**95% CI**	***p*** **-value**
**DeLong test**						
Nomogram:APS3	4.700		<0.001	4.025		<0.001
Nomogram:OASIS	6.169		<0.001	3.453		<0.001
**Categorical NRI**						
Nomogram:APS3	0.351	0.269–0.423		0.419	0.315–0.527	
Nomogram:OASIS	0.279	0.216–0.343		0.206	0.102–0.324	
**Continuous NRI**						
Nomogram:APS3	0.543	0.455–0.649		0.564	0.438–0.713	
Nomogram:OASIS	0.500	0.388–0.612		0.417	0.219–0.575	
**IDI**						
Nomogram:APS3	0.103	0.074–0.147	<0.001	0.132	0.075–0.201	<0.001
Nomogram:OASIS	0.103	0.066–0.141	<0.001	0.121	0.065–0.187	<0.001

### Clinical application of nomograms

**Figure 4:**
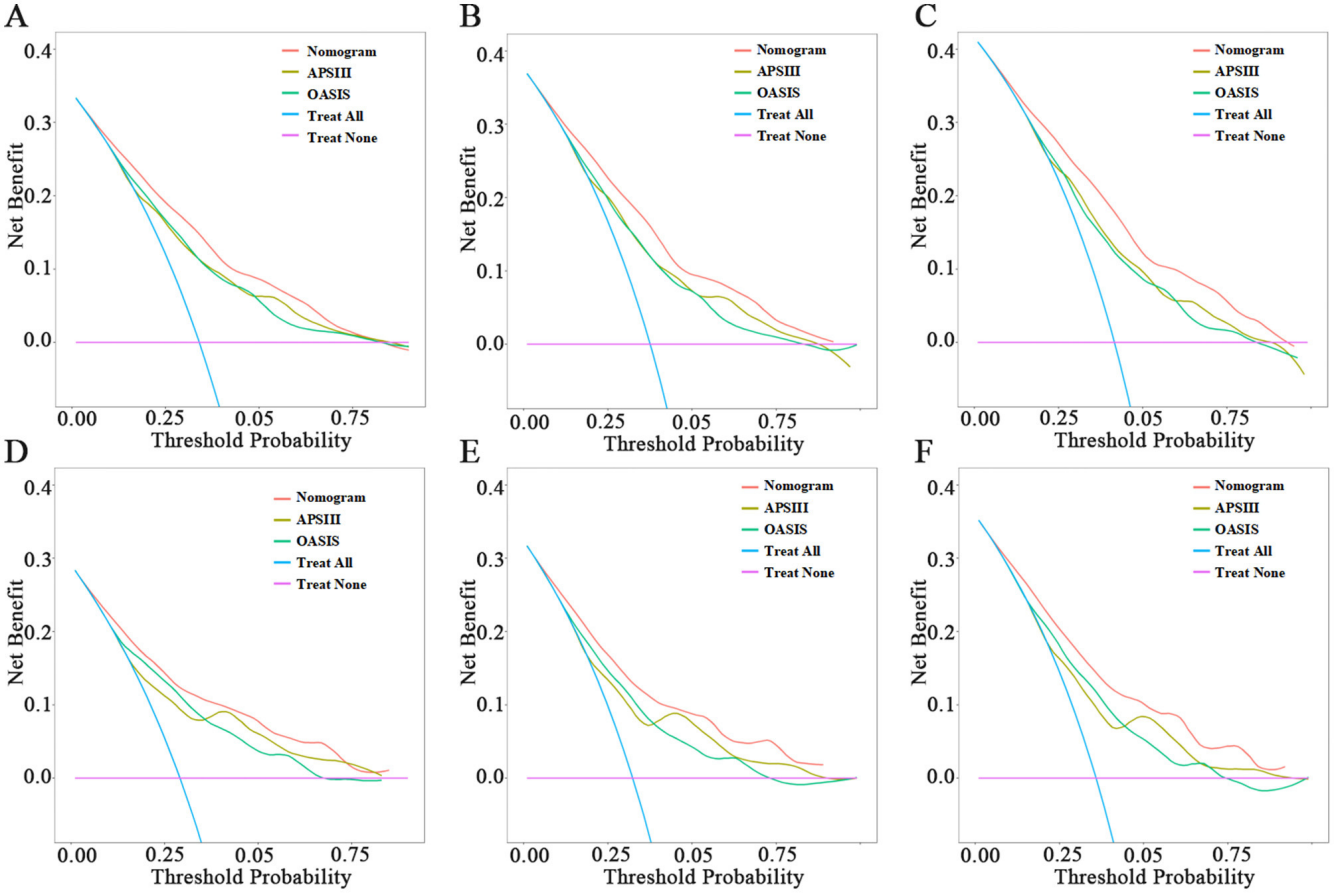
Decision-curve analysis of nomogram. **(A–C)** The DCA curves of 3-month, 6-month, and 1-year in the training set, respectively. **(D–F)** The DCA curves of 3-month, 6-month, and 1-year in the testing set, respectively.

**Figure 5:**
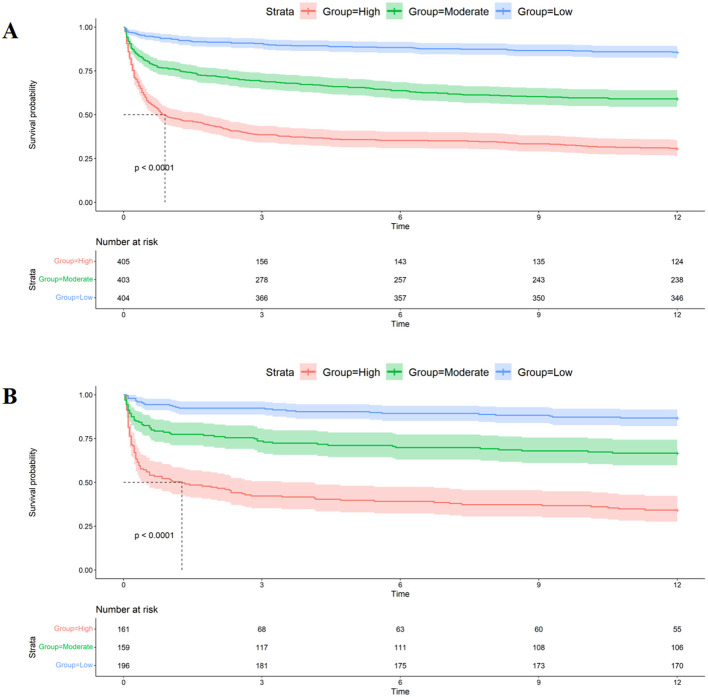
Kaplan-Meier curves of nomogram. **(A)** The Kaplan-Meier curves of nomogram in the training set. **(B)** The Kaplan-Meier curves of nomogram in the testing set.

When assessing clinical utility, DCA was used to compare the clinical benefits of the nomogram with APSIII and OASIS scores (Figure 4). Compared to APSIII and OASIS scores, the nomogram provided better predictive ability when the threshold probability was between 5 and 90% in the training set and threshold probability >5% in the testing set (3-month: training set 5–75%, testing set 5–70%; 6-month: training set 5–90%, testing set >5%). The Kaplan-Meier curves also indicated that the nomogram could effectively identify low-risk, medium-risk, and high-risk patients (Figure 5).

## Discussion

There Numerous studies have focused on predicting mortality rates for patients with ICH in the ICU, primarily concentrating on in-hospital or 30-day survival, but long-term survival is also significant (14). In this study, we developed and validated a nomogram for predicting long-term survival after ICU discharge in ICH patients.

We adjusted for known predictors of mortality after ICH and identified PWR as an independent prognostic factor for 3-, 6-month, and 1-year mortality in ICH patients, as well as for concurrent infection, kidney injury, brain edema, and hydrocephalus during ICU stay. Using LASSO and multivariate COX regression, we identified eight independent prognostic factors for 1-year survival: PWR, age, weight, GCS score, mechanical ventilation, glucose, RBC, and BUN. These variables were then used to construct a nomogram for assessing long-term survival in ICH patients.

Nomograms are widely used in clinical diagnosis and prognosis assessment. APSIII and OASIS are commonly used severity scores in the ICU, incorporating laboratory indicators within 24 h of ICU admission (15). One study demonstrated that higher acute physiology scores upon admission are independently associated with 1-year survival after ICU discharge and can serve as predictive scores for long-term prognosis in ICU patients (16). The ICH score, the most widely used clinical short-term prediction scale for ICH, has been validated for assessing 1-year mortality and functional outcomes following acute ICH (17, 18). Therefore, APSIII, OASIS, and ICH scores were chosen for comparison.

The effectiveness of the nomogram was confirmed through calibration curves, AUC, Delong test, NRI, IDI, and DCA. Previous studies have shown that NRI, IDI, and DCA are reliable measures for assessing predictive models (19–21). Our findings align with this, demonstrating that the new nomogram improves risk stratification and aids clinicians in targeting treatments more effectively. The categorical NRI values confirm that the nomogram enhances patient risk classification compared to traditional scoring systems, enabling better identification of high-risk patients. Additionally, the continuous NRI and IDI further support the nomogram's predictive performance, enhancing its ability to distinguish between survival and non-survival outcomes. These improvements underscore the nomogram's clinical utility, as demonstrated by DCA, which highlights its practical benefits for risk assessment and decision-making.

This study is the first to examine the relationship between PWR and 3-, 6-month, and 1-year mortality risk in ICU patients with ICH. The model constructed with PWR demonstrates better variable usability and predictive performance compared to commonly used scoring systems. By providing a quick and reliable method for assessing patient prognosis, the nomogram can be easily integrated into daily clinical workflows, helping clinicians prioritize resources, decide on intervention intensity, and engage in more informed discussions with patients and their families about expected outcomes.

Age is an independent prognostic factor for in-hospital and long-term mortality in ICH patients, as confirmed by most studies (22). Our study found that older patients face a higher 1-year mortality risk, with patients aged 58–80 and over 80 having a 139.0 and 285.4% higher risk, respectively, compared to those under 58. Weight is closely linked to mortality rates in ICH patients. Previous studies have shown that underweight patients have a higher mortality risk, while overweight or obese patients have a reduced long-term mortality risk (23, 24). These findings suggest that patients' obesity status should be considered as an indicator of metabolic reserve capacity and viability. In our study, every 1-unit increase in weight resulted in a 0.9% decrease in 1-year mortality risk, indicating that patients with higher weight had a relatively lower mortality risk.

Consistent with other studies, we found that GCS scores and the need for mechanical ventilation during the ICU stay were prognostic factors for ICH patient mortality. GCS scores have strong predictive value for both short-term and long-term mortality in ICH patients (25, 26). Our study revealed that for every one-point increase in GCS score, the 1-year mortality risk decreased by 8.9%, indicating that patients with higher GCS scores had a relatively lower mortality risk. Mechanical ventilation is often necessary for ICH patients in the ICU to manage respiratory rhythm abnormalities and maintain appropriate oxygenation and carbon dioxide levels. However, patients on mechanical ventilation may have higher rates of infection and mortality (27, 28). Our study confirms that ICH patients requiring mechanical ventilation have a 73.4% higher 1-year mortality risk than those who do not. This emphasizes the importance of carefully considering the timing and indications of mechanical ventilation as a prognostic factor for long-term mortality after ICH.

In our nomogram, we incorporated laboratory indicators such as glucose, BUN, RBC count, and PWR, which are straightforward and readily obtainable. Higher levels of BUN and blood glucose correlate with poorer prognosis. BUN, a byproduct of protein metabolism, serves as a clinical biomarker of kidney function. Recent studies have demonstrated that elevated BUN levels are linked to poor outcomes in various diseases (29, 30). Similarly, high blood glucose levels have been connected to unfavorable functional outcomes following ICH (31). In our nomogram, abnormal blood glucose levels in ICH patients are a risk factor for 1-year mortality, with each one-unit increase in blood glucose increasing the risk of 1-year mortality by 2.9%. This suggests that elevated blood glucose levels within the first 24 h are associated with a higher mortality risk.

RBC-related indicators, such as hemoglobin and red cell distribution width (RDW), have been linked to post-stroke mortality, clinical outcomes, and functional recovery (32). Lower RBC levels might indicate impaired brain oxygenation capacity, and red blood cell transfusion can improve survival rates in ICH patients (33). In our study, we included RBC-related indicators, such as hemoglobin and RBC count, but only RBC count was included in the final nomogram. Multivariate Cox regression analysis showed that for every one-unit increase in RBC count, the risk of 1-year mortality decreased by 21%. Thus, within a certain range, a higher red blood cell count is associated with a relatively lower risk of death.

PWR is a newly identified inflammatory biomarker (11). Following ICH, white blood cells play a crucial role in the body's immune response and inflammatory regulation (34). Higher white blood cell counts usually indicate a more severe inflammatory response (35). Lower platelet counts in ICH patients may suggest reduced coagulation function, leading to a poorer prognosis (36). PWR, calculated as the ratio of platelet count to white blood cell count, captures both aspects. A higher PWR value may indicate a milder inflammatory response and better coagulation function, making it a useful indicator for predicting the prognosis of ICH. Patients with PWR >14.846 had a 41.7% lower 1-year mortality risk compared to those with PWR <14.846, indicating a better prognosis for higher PWR values.

In summary, we developed a nomogram that includes PWR to evaluate its role in predicting the prognosis of ICH patients after ICU discharge. This nomogram offers clinicians a practical tool for assessing the survival rates of ICH patients at 3, 6 months, and 1 year. However, there are limitations to our study. Firstly, the study is based on a single-center retrospective design with a relatively small sample size, which may introduce patient selection bias. Thus, our findings require validation through larger-scale multicenter studies. Additionally, while we assessed 1-year mortality, the exact timing (whether during ICU stay or after discharge) was not recorded. Future studies should differentiate between these time points to better understand PWR's long-term prognostic value. Finally, while our study focused on PWR and its predictive value for long-term mortality in ICH patients, there are other emerging biomarkers and pathways that warrant exploration in future research. Recent studies have highlighted the potential role of neutrophil extracellular traps (NETs), fibrinogen, and pro-inflammatory cytokines such as IL-6 and TNF-α in the progression of ICH (37–39). Incorporating these additional factors may improve the model's ability to predict patient prognosis and guide clinical decision-making.

## Conclusion

In conclusion, we established a predictive nomogram to assess the long-term survival of ICH patients after ICU discharge based on several clinical and laboratory parameters, including the newly identified inflammatory marker PWR. Our study showed that PWR is a useful indicator for predicting the prognosis of ICH, and our nomogram achieved good predictive performance compared to commonly used scales in the ICU.

## Data Availability

The original contributions presented in the study are included in the article/Supplementary material, further inquiries can be directed to the corresponding author.
