# Supplementary material for: Incorporating platelet-to-white blood cell ratio into survival prediction models for intracerebral hemorrhage: a nomogram approach
Source: Front Neurol. 2024 Oct 10;15:1464216. doi: 10.3389/fneur.2024.1464216 (PMC11499137; doi:10.3389/fneur.2024.1464216)
Supplement: Supplementary file 1 [file Data_Sheet_1.docx]

Supplementary Material

# Supplementary Figures and Tables

## Supplementary Figures


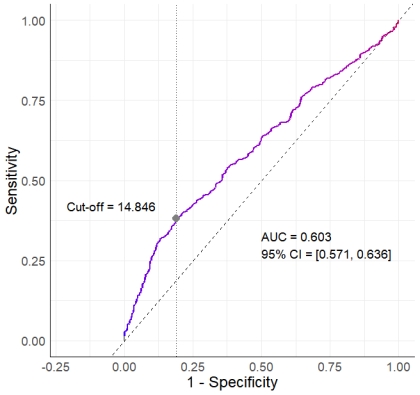


**Supplementary figure 1. ROC curves of the first day PWR for predicting 1-Year mortality.**


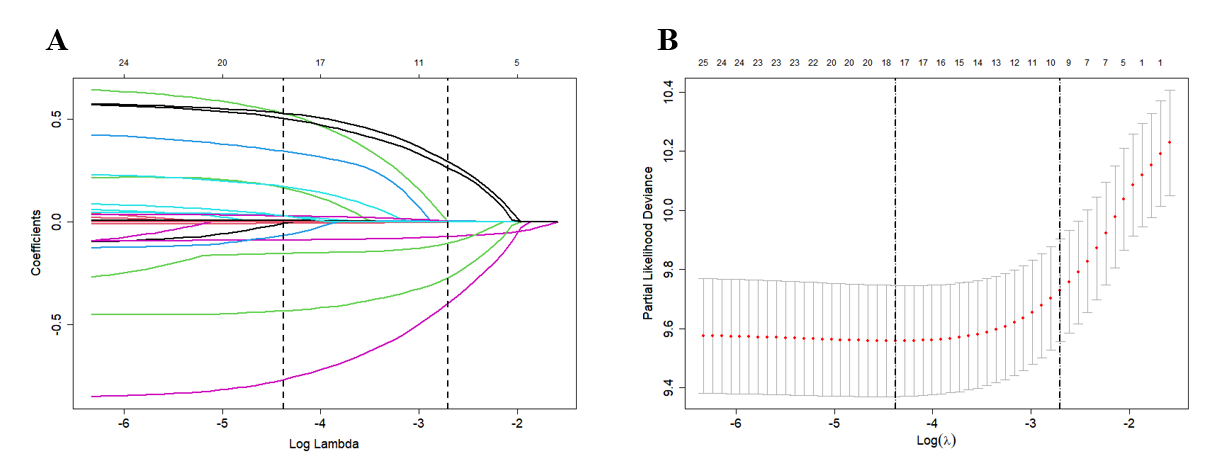


**Supplementary figure 2. LASSO regression analysis results.**

1. The LASSO path. B. The coefficient estimates of each variable against log(Lambda) values.


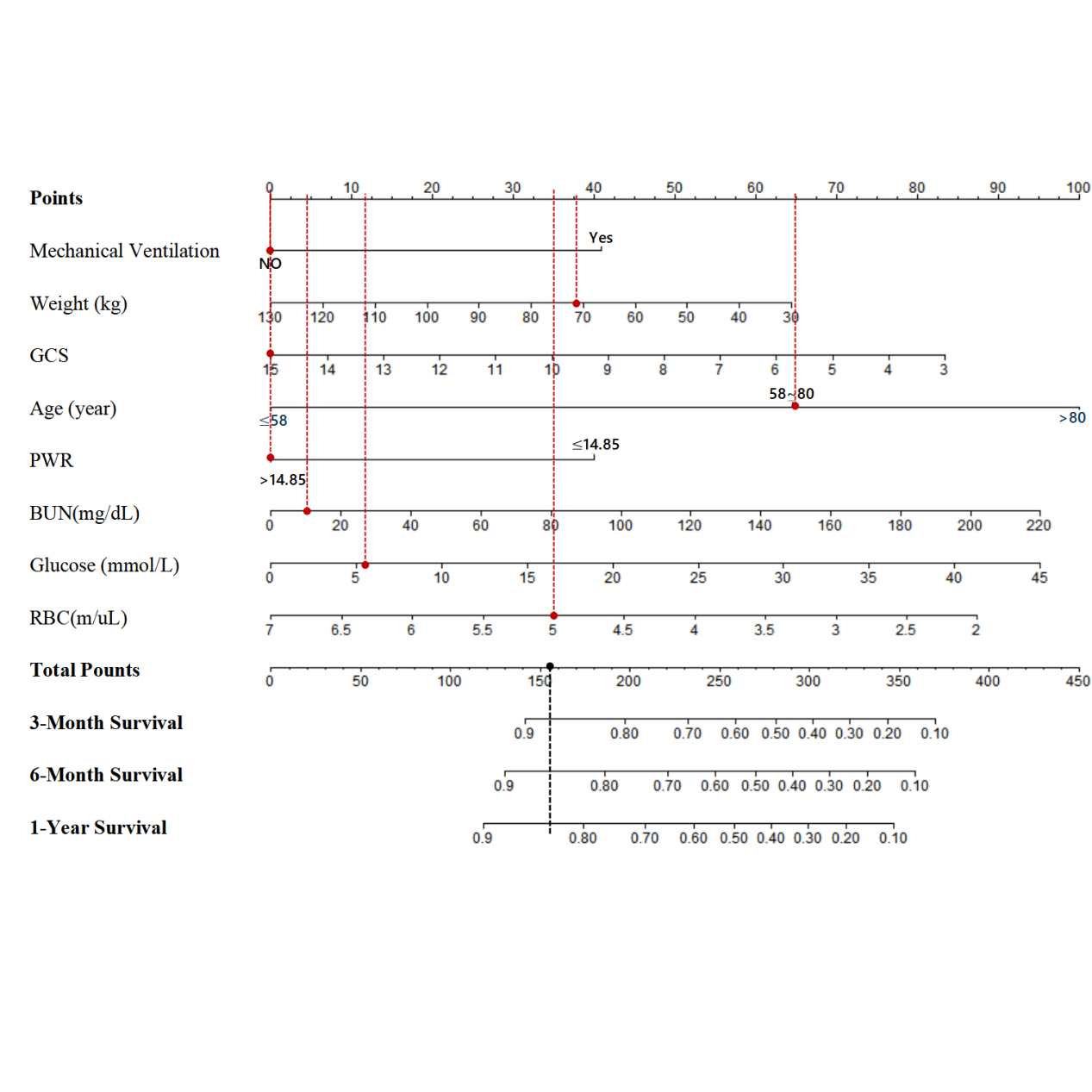


**Supplementary figure 3. An example of the nomogram**

GCS, Glasgow Coma Scale; PWR, platelet-to-white blood cell ratio; BUN, blood urea nitrogen; RBC, red blood cell. An example: a 60-year-old patient with a weight of 72kg had the following laboratory values: RBC count of 5m/μL, platelet count of 207 k/μL, WBC count of 10.4 × 10^3/μL, PWR value of 19.9, glucose level of 5.5 mmol/L, and BUN of 10 mg/dL. The patient had a GCS score of 15 and did not receive mechanical ventilation. Based on the nomogram, the total score was 154.1, and the predicted probabilities of survival at 3 months, 6 months, and 1 year were all above 80%, indicating a high survival rate that is consistent with the patient's actual outcome.


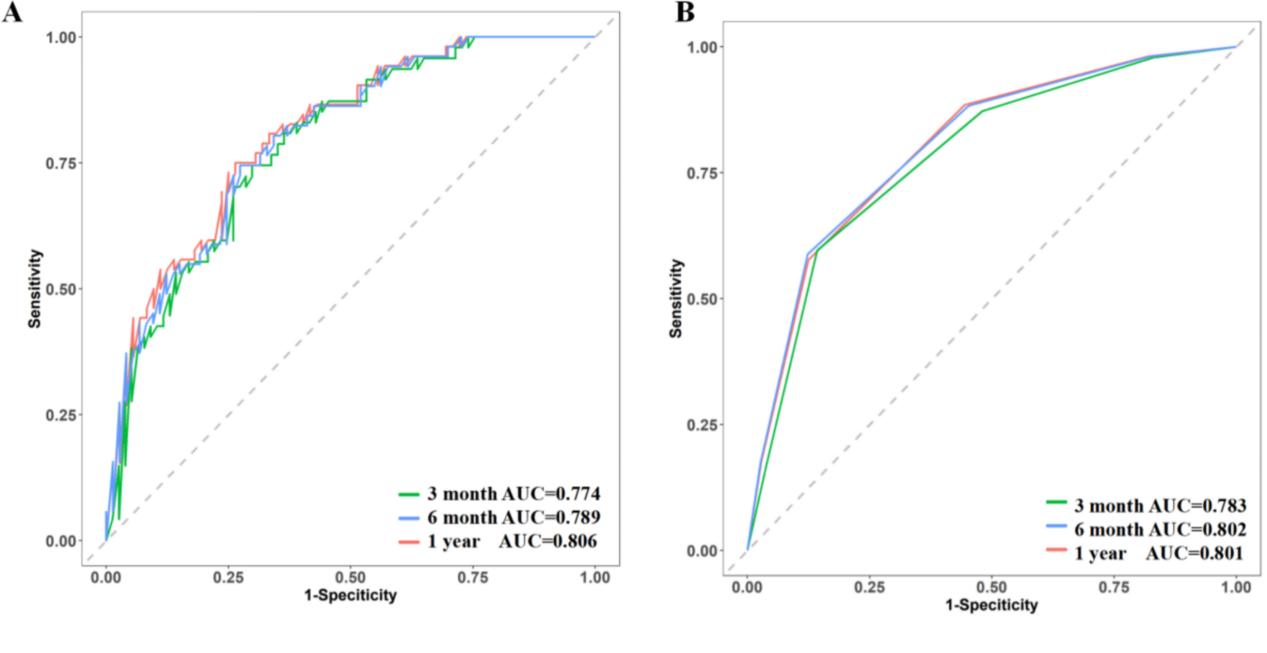


**Supplementary figure 4. Evaluation of the Nomogram and ICH score in the Imaging testing sets**

A: The ROC curves of Nomogram. B: The ROC curves of ICH score.

## Supplementary Tables

**Supplementary table 1. The patient characteristic of training set and testing set.**

| **Variables** | **Total  (N=1728)** | **Train  (N=1212)** | **Test  (N=516)** | **p value** |
| --- | --- | --- | --- | --- |
| **Age, year** | 70.1 [58.3;80.9] | 70.4 [58.0;80.9] | 69.5 [58.4;80.8] | 0.609 |
| **Weight, kg** | 76.6 [64.3;89.0] | 76.8 [64.6;88.7] | 75.2 [64.0;89.4] | 0.678 |
| **Gender, n (%)** |  |  |  | 0.569 |
| Male | 944 (54.6%) | 668 (55.1%) | 276 (53.5%) |  |
| Female | 784 (45.4%) | 544 (44.9%) | 240 (46.5%) |  |
| **Ethnicity, n (%)** |  |  |  | 0.827 |
| White | 1043 (60.4%) | 737 (60.8%) | 306 (59.3%) |  |
| Black | 163 (9.43%) | 114 (9.41%) | 49 (9.50%) |  |
| Other | 522 (30.2%) | 361 (29.8%) | 161 (31.2%) |  |
| **Alcohol dependence, n (%)** | 155 (8.97%) | 107 (8.83%) | 48 (9.30%) | 0.823 |
| **Smoking, n (%)** | 511 (29.6%) | 333 (27.5%) | 178 (34.5%) | 0.004 |
| **Mechanical Ventilation** | 704 (40.7%) | 508 (41.9%) | 196 (38.0%) | 0.142 |
| **Comorbidities, n (%)** |  |  |  |  |
| Heart disease | 317 (18.3%) | 220 (18.2%) | 97 (18.8%) | 0.803 |
| Chronic pulmonary disease | 228 (13.2%) | 159 (13.1%) | 69 (13.4%) | 0.948 |
| Rheumatic disease | 39 (2.26%) | 29 (2.39%) | 10 (1.94%) | 0.685 |
| Diabetes | 415 (24.0%) | 293 (24.2%) | 122 (23.6%) | 0.861 |
| Renal disease | 194 (11.2%) | 140 (11.6%) | 54 (10.5%) | 0.568 |
| Malignant cancer | 169 (9.78%) | 127 (10.5%) | 42 (8.14%) | 0.159 |
| Liver disease | 94 (5.44%) | 70 (5.78%) | 24 (4.65%) | 0.408 |
| Hypertension | 1345 (77.8%) | 932 (76.9%) | 413 (80.0%) | 0.169 |
| **Scores** |  |  |  |  |
| GCS | 11.0 [7.00;14.0] | 11.0 [7.00;14.0] | 12.0 [7.00;14.0] | 0.269 |
| APSiii | 40.0 [29.0;55.0] | 41.0 [29.0;56.0] | 38.5 [28.8;53.0] | 0.085 |
| OASIS | 33.0 [27.0;40.0] | 33.0 [27.0;40.0] | 33.0 [26.0;40.0] | 0.484 |
| **Complication, n (%)** |  |  |  |  |
| Cerebral edema | 741 (42.9%) | 521 (43.0%) | 220 (42.6%) | 0.935 |
| Hydrocephalus | 284 (16.4%) | 206 (17.0%) | 78 (15.1%) | 0.371 |
| Nosocomial infections | 871 (50.4%) | 617 (50.9%) | 254 (49.2%) | 0.557 |
| Kidney failure | 253 (14.6%) | 182 (15.0%) | 71 (13.8%) | 0.547 |
| **Laboratory events** |  |  |  |  |
| PWR | 19.0 [14.5;24.4] | 19.0 [14.5;24.4] | 19.1 [14.3;24.3] | 0.816 |
| Hemoglobin, g/dL | 12.9 [11.7;14.2] | 12.9 [11.7;14.2] | 12.9 [11.8;14.2] | 0.491 |
| Platelets, K/uL | 215 [171;268] | 216 [173;268] | 214 [170;273] | 0.879 |
| WBC, K/uL | 11.3 [8.80;14.9] | 11.3 [8.80;14.9] | 11.4 [8.70;14.9] | 0.843 |
| RBC, m/uL | 4.29 [3.85;4.69] | 4.28 [3.85;4.67] | 4.32 [3.87;4.72] | 0.200 |
| BUN, mg/dL | 18.0 [13.0;24.0] | 18.0 [14.0;24.0] | 17.0 [13.0;23.0] | 0.158 |
| Creatinine, mg/dL | 0.90 [0.80;1.20] | 0.90 [0.80;1.20] | 0.90 [0.80;1.20] | 0.84 |
| Glucose, mmol/L | 8.00 [6.61;9.89] | 8.00 [6.61;9.94] | 7.94 [6.56;9.83] | 0.748 |
| PT, s | 12.7 [11.7;14.6] | 12.8 [11.8;14.7] | 12.6 [11.7;14.3] | 0.075 |
| APTT, s | 28.9 [26.1;32.6] | 28.9 [26.1;32.7] | 28.8 [26.2;32.6] | 0.908 |
| **1-Year Status (%)** |  |  |  | 0.030 |
| Survival | 1039 (60.1%) | 708 (58.4%) | 331 (64.1%) |  |
| dead | 689 (39.9%) | 504 (41.6%) | 185 (35.9%) |  |
| **Length of hospital stay, days** | 8.56 [4.71;15.6] | 8.85 [4.78;16.0] | 8.02 [4.53;14.3] | 0.061 |
| **Length of ICU stay, days** | 3.76 [2.02;7.91] | 3.80 [2.04;8.31] | 3.58 [1.98;7.14] | 0.119 |

**Supplementary table 2. The baseline clinical characteristics of the training set Patients** .

| **Variables** | **Total  (N=1212)** | **1-Year survival (N=708, 58.4%)** | **1-Year non-survival (N=504, 41.6%)** | **p-value** |
| --- | --- | --- | --- | --- |
| **Age, year** | 70.4 [58.0;80.9] | 65.5 [54.4;76.7] | 75.9 [64.9;84.9] | **<0.001** |
| **Age group** |  |  |  | **<0.001** |
| ≤58 | 303 (25.0%) | 239 (33.8%) | 64 (12.7%) |  |
| 59-80 | 581 (47.9%) | 335 (47.3%) | 246 (48.8%) |  |
| >80 | 328 (27.1%) | 134 (18.9%) | 194 (38.5%) |  |
| **Weight, kg** | 76.8 [64.6;88.7] | 79.0 [67.9;91.0] | 73.6 [61.4;85.0] | **<0.001** |
| **Gender, n (%)** |  |  |  | **0.043** |
| Male | 668 (55.1%) | 408 (57.6%) | 260 (51.6%) |  |
| Female | 544 (44.9%) | 300 (42.4%) | 244 (48.4%) |  |
| **Ethnicity, n (%)** |  |  |  | 0.599 |
| White | 737 (60.8%) | 438 (61.9%) | 299 (59.3%) |  |
| Black | 114 (9.41%) | 67 (9.46%) | 47 (9.33%) |  |
| Other | 361 (29.8%) | 203 (28.7%) | 158 (31.3%) |  |
| **Alcohol dependence, n (%)** | 107 (8.83%) | 56 (7.91%) | 51 (10.1%) | 0.217 |
| **Smoking, n (%)** | 333 (27.5%) | 202 (28.5%) | 131 (26.0%) | 0.362 |
| **Mechanical Ventilation** | 508 (41.9%) | 228 (32.2%) | 280 (55.6%) | **<0.001** |
| **Comorbidities, n (%)** |  |  |  |  |
| Heart disease | 220 (18.2%) | 105 (14.8%) | 115 (22.8%) | **0.001** |
| Chronic pulmonary disease | 159 (13.1%) | 82 (11.6%) | 77 (15.3%) | 0.073 |
| Rheumatic disease | 29 (2.39%) | 20 (2.82%) | 9 (1.79%) | 0.329 |
| Diabetes | 293 (24.2%) | 163 (23.0%) | 130 (25.8%) | 0.297 |
| Renal disease | 140 (11.6%) | 62 (8.76%) | 78 (15.5%) | **<0.001** |
| Malignant cancer | 127 (10.5%) | 54 (7.63%) | 73 (14.5%) | **<0.001** |
| Liver disease | 70 (5.78%) | 26 (3.67%) | 44 (8.73%) | **<0.001** |
| Hypertension | 932 (76.9%) | 533 (75.3%) | 399 (79.2%) | 0.131 |
| **Scores** |  |  |  |  |
| GCS | 11.0 [7.00;14.0] | 13.0 [9.00;14.0] | 9.00 [6.00;13.0] | **<0.001** |
| APSiii | 41.0 [29.0;56.0] | 35.0 [26.0;46.2] | 50.0 [37.8;71.0] | **<0.001** |
| OASIS | 33.0 [27.0;40.0] | 31.0 [25.0;36.0] | 37.0 [32.0;43.0] | **<0.001** |
| **Complication, n (%)** |  |  |  |  |
| Cerebral edema | 521 (43.0%) | 291 (41.1%) | 230 (45.6%) | 0.130 |
| Hydrocephalus | 206 (17.0%) | 106 (15.0%) | 100 (19.8%) | **0.032** |
| Nosocomial infections | 617 (50.9%) | 318 (44.9%) | 299 (59.3%) | **<0.001** |
| Kidney failure | 182 (15.0%) | 88 (12.4%) | 94 (18.7%) | **0.004** |
| **Laboratory events** |  |  |  |  |
| PWR | 19.0 [14.5;24.4] | 20.0 [15.9;25.6] | 17.4 [12.5;22.8] | **<0.001** |
| Hemoglobin, g/dL | 12.9 [11.7;14.2] | 13.1 [12.0;14.4] | 12.6 [11.2;13.8] | **<0.001** |
| Platelets, K/uL | 216 [173;268] | 222 [183;269] | 204 [162;261] | **<0.001** |
| WBC, K/uL | 11.3 [8.80;14.9] | 11.0 [8.50;14.2] | 12.1 [9.28;15.7] | **0.001** |
| RBC, m/uL | 4.28 [3.85;4.67] | 4.38 [4.00;4.78] | 4.12 [3.69;4.55] | **<0.001** |
| BUN, mg/dL | 18.0 [14.0;24.0] | 17.0 [13.0;22.0] | 20.0 [15.0;27.2] | **<0.001** |
| Creatinine, mg/dL | 0.90 [0.80;1.20] | 0.90 [0.80;1.10] | 1.00 [0.80;1.30] | **0.001** |
| Glucose, mmol/L | 8.00 [6.61;9.94] | 7.56 [6.39;9.17] | 8.61 [7.17;10.7] | **<0.001** |
| PT, s | 12.8 [11.8;14.7] | 12.5 [11.7;14.1] | 13.2 [12.0;15.6] | **<0.001** |
| APTT,s | 28.9 [26.1;32.7] | 28.6 [26.1;31.8] | 29.5 [25.9;33.9] | **0.031** |
| **Length of hospital stay, days** | 8.85 [4.78;16.0] | 9.77 [5.48;17.4] | 6.91 [3.70;14.0] | **<0.001** |
| **Length of ICU stay, days** | 3.80 [2.04;8.31] | 3.84 [2.00;8.33] | 3.70 [2.09;8.30] | 0.954 |

**Supplementary table 3. Characteristics of participants categorized by PWR.**

| **Variables** | **Total  (N=1212)** | **Low PWR≤14.846 (N=327, 27.0%)** | **High PWR>14.846 (N=885, 73.0%)** | **p value** |
| --- | --- | --- | --- | --- |
| **Age, year** | 70.4 [58.0;80.9] | 69.5 [56.9;79.3] | 70.6 [58.3;81.5] | 0.094 |
| **Age group** |  |  |  | 0.300 |
| ≤58 | 303 (25.0%) | 87 (26.6%) | 216 (24.4%) |  |
| 59-80 | 581 (47.9%) | 162 (49.5%) | 419 (47.3%) |  |
| >80 | 328 (27.1%) | 78 (23.9%) | 250 (28.2%) |  |
| **Weight, kg** | 76.8 [64.6;88.7] | 78.2 [67.0;87.7] | 76.2 [64.0;89.0] | 0.176 |
| **Gender, n (%)** |  |  |  | **0.017** |
| Male | 668 (55.1%) | 199 (60.9%) | 469 (53.0%) |  |
| Female | 544 (44.9%) | 128 (39.1%) | 416 (47.0%) |  |
| **Ethnicity, n (%)** |  |  |  | **0.001** |
| White | 737 (60.8%) | 185 (56.6%) | 552 (62.4%) |  |
| Black | 114 (9.41%) | 20 (6.12%) | 94 (10.6%) |  |
| Other | 361 (29.8%) | 122 (37.3%) | 239 (27.0%) |  |
| **Alcohol dependence, n (%)** | 107 (8.83%) | 39 (11.9%) | 68 (7.68%) | **0.028** |
| **Smoking, n (%)** | 333 (27.5%) | 86 (26.3%) | 247 (27.9%) | 0.628 |
| **Mechanical Ventilation:** | 508 (41.9%) | 208 (63.6%) | 300 (33.9%) | **<0.001** |
| **Comorbidities, n (%)** |  |  |  |  |
| Heart disease | 220 (18.2%) | 60 (18.3%) | 160 (18.1%) | 0.981 |
| Chronic pulmonary disease | 159 (13.1%) | 47 (14.4%) | 112 (12.7%) | 0.490 |
| Rheumatic disease | 29 (2.39%) | 6 (1.83%) | 23 (2.60%) | 0.575 |
| Diabetes | 293 (24.2%) | 75 (22.9%) | 218 (24.6%) | 0.591 |
| Renal disease | 140 (11.6%) | 40 (12.2%) | 100 (11.3%) | 0.726 |
| Malignant cancer | 127 (10.5%) | 40 (12.2%) | 87 (9.83%) | 0.269 |
| Liver disease | 70 (5.78%) | 35 (10.7%) | 35 (3.95%) | **<0.001** |
| Hypertension | 932 (76.9%) | 241 (73.7%) | 691 (78.1%) | 0.126 |
| **Scores** |  |  |  |  |
| GCS | 11.0 [7.00;14.0] | 9.00 [6.00;13.0] | 12.0 [8.00;14.0] | **<0.001** |
| APSiii | 41.0 [29.0;56.0] | 48.0 [35.0;68.0] | 39.0 [28.0;51.0] | **<0.001** |
| OASIS | 33.0 [27.0;40.0] | 36.0 [31.0;43.0] | 32.0 [26.0;38.0] | **<0.001** |
| **Complication, n (%)** |  |  |  |  |
| Cerebral edema | 521 (43.0%) | 152 (46.5%) | 369 (41.7%) | 0.153 |
| Hydrocephalus | 206 (17.0%) | 75 (22.9%) | 131 (14.8%) | **0.001** |
| Nosocomial infections | 617 (50.9%) | 205 (62.7%) | 412 (46.6%) | **<0.001** |
| Kidney failure | 182 (15.0%) | 72 (22.0%) | 110 (12.4%) | **<0.001** |
| **Laboratory events/Vital signs** |  |  |  |  |
| PWR | 19.0 [14.5;24.4] | 11.9 [9.95;13.4] | 21.5 [18.2;26.9] | **<0.001** |
| Hemoglobin, g/dL | 12.9 [11.7;14.2] | 12.9 [11.5;14.3] | 12.9 [11.7;14.1] | 0.962 |
| Platelets, K/uL | 216 [173;268] | 174 [140;209] | 232 [190;283] | **<0.001** |
| WBC, K/uL | 11.3 [8.80;14.9] | 15.3 [12.4;18.6] | 10.3 [8.10;12.8] | **<0.001** |
| RBC, m/uL | 4.28 [3.85;4.67] | 4.21 [3.74;4.66] | 4.30 [3.90;4.68] | **0.030** |
| BUN, mg/dL | 18.0 [14.0;24.0] | 20.0 [14.0;27.0] | 18.0 [13.0;23.0] | **<0.001** |
| Creatinine, mg/dL | 0.90 [0.80;1.20] | 1.00 [0.80;1.30] | 0.90 [0.80;1.20] | **<0.001** |
| Glucose, mmol/L | 8.00 [6.61;9.94] | 8.94 [7.36;11.1] | 7.67 [6.50;9.28] | **<0.001** |
| PT, s | 12.8 [11.8;14.7] | 13.2 [12.0;16.2] | 12.6 [11.7;14.3] | **<0.001** |
| APTT, s | 28.9 [26.1;32.7] | 28.7 [25.9;33.3] | 29.0 [26.2;32.5] | 0.796 |
| **1-Year Status, n (%)** |  |  |  | **<0.001** |
| Survival | 708 (58.4%) | 135 (41.3%) | 573 (64.7%) |  |
| dead | 504 (41.6%) | 192 (58.7%) | 312 (35.3%) |  |
| **Length of hospital stay, days** | 8.85 [4.78;16.0] | 10.5 [4.47;20.2] | 8.48 [4.89;14.8] | 0.050 |
| **Length of ICU stay, days** | 3.80 [2.04;8.31] | 4.88 [2.34;12.2] | 3.62 [1.96;7.24] | **<0.001** |

**Supplementary table 4. Evaluation of the nomogram in the training and testing sets for second outcome**

| Test | Training Set | | | Testing Set | | |
| --- | --- | --- | --- | --- | --- | --- |
|  | Estimate  (Z for DeLong test) | 95% CI | p-  value | Estimate  (Z for DeLong test) | 95% CI | p-  value |
| **DeLong test** |  |  |  |  |  |  |
| 3month-  Nomogram:APS3 | 4.030 |  | <0.001 | 2.940 |  | 0.003 |
| 3month-  Nomogram:OASIS | 4.254 |  | <0.001 | 2.231 |  | 0.026 |
| 6month-  Nomogram:APS3 | 4.180 |  | <0.001 | 3.554 |  | <0.001 |
| 6month-  Nomogram:OASIS | 5.156 |  | <0.001 | 3.173 |  | 0.002 |
| **Categorical NRI** |  |  |  |  |  |  |
| 3month-  Nomogram:APS3 | 0.270 | 0.196-0.351 |  | 0.273 | 0.155-0.394 |  |
| 3month-  Nomogram:OASIS | 0.168 | 0.088-0.245 |  | 0.130 | 0.014-0.234 |  |
| 6month-  Nomogram:APS3 | 0.374 | 0.294-0.449 |  | 0.352 | 0.234-0.468 |  |
| 6month-  Nomogram:OASIS | 0.243 | 0.172-0.328 |  | 0.181 | 0.087-0.289 |  |
| **Continuous NRI** |  |  |  |  |  |  |
| 3month-  Nomogram:APS3 | 0.481 | 0.365-0.607 |  | 0.503 | 0.317-0.656 |  |
| 3month-  Nomogram:OASIS | 0.442 | 0.349-0.546 |  | 0.378 | 0.224-0.564 |  |
| 6month-  Nomogram:APS3 | 0.506 | 0.405-0.593 |  | 0.552 | 0.398-0.705 |  |
| 6month-  Nomogram:OASIS | 0.462 | 0.368-0.553 |  | 0.423 | 0.278-0.579 |  |
| **IDI** |  |  |  |  |  |  |
| 3month-  Nomogram:APS3 | 0.083 | 0.051-0.118 | <0.001 | 0.111 | 0.058- 0.192 | 0.01 |
| 3month-  Nomogram:OASIS | 0.081 | 0.052-0.117 | <0.001 | 0.111 | 0.065-0.179 | <0.001 |
| 6month-  Nomogram:APS3 | 0.091 | 0.052-0.137 | <0.001 | 0.121 | 0.040-0.201 | <0.001 |
| 6month-  Nomogram:OASIS | 0.093 | 0.069-0.124 | <0.001 | 0.119 | 0.094-0.181 | <0.001 |

**Supplementary table 5. Evaluation of the nomogram and ICH score in the Imaging testing sets**

| **DeLong test**  Nomogram:ICH score | **Estimate**  **(Z for DeLong test)** | **p-value** |
| --- | --- | --- |
| 3 month | -0.23558 | 0.8138 |
| 6 month | -0.32649 | 0.7441 |
| 1 year | 0.14149 | 0.8875 |
